# Sedentary behaviours during pregnancy: a systematic review

**DOI:** 10.1186/s12966-017-0485-z

**Published:** 2017-03-16

**Authors:** Caterina Fazzi, David H. Saunders, Kathryn Linton, Jane E. Norman, Rebecca M. Reynolds

**Affiliations:** 1 Tommy’s Centre for Maternal and Fetal Health, MRC/University of Edinburgh, Centre for Reproductive Health, University of Edinburgh, Queen’s Medical Research Institute, 47 Little France Crescent, Edinburgh, EH16 4TJ United Kingdom; 2 Department of Physical Education, Sports and Recreation, Metropolitan University of Educational Sciences, Santiago, Chile; 3 Physical Activity for Health Research Centre (PAHRC), Institute for Sport, Physical Education and Health Sciences, University of Edinburgh, Holyrood Road, Edinburgh, EH8 8AQ United Kingdom; 4 Metabolic Unit Western General Hospital, Crewe Road South, Edinburgh, EH4 2XE United Kingdom; 5 University BHF Centre for Cardiovascular Science, University of Edinburgh, Edinburgh, United Kingdom

**Keywords:** Sedentary behaviours, Sedentarism, Pregnancy

## Abstract

**Background:**

In the general population, at least 50% of time awake is spent in sedentary behaviours. Sedentary behaviours are activities that expend less energy than 1.5 metabolic equivalents, such as sitting. The amount of time spent in sedentary behaviours is a risk factor for diseases such as type 2 diabetes, cardiovascular disease, and death from all causes. Even individuals meeting physical activity guidelines are at a higher risk of premature death and adverse metabolic outcomes if they sit for extended intervals. The associations between sedentary behaviour with type 2 diabetes and with impaired glucose tolerance are stronger for women than for men. It is not known whether sedentary behaviour in pregnancy influences pregnancy outcomes, but if those negative outcomes observed in general adult population also occur in pregnancy, this could have implications for adverse outcomes for mothers and offspring.

We aimed to determine the proportion of time spent in sedentary behaviours among pregnant women, and the association of sedentary behaviour with pregnancy outcomes in mothers and offspring.

**Methods:**

Two researchers independently performed the literature search using 5 different electronic bibliographic databases. Studies were included if sedentary behaviours were assessed during pregnancy. Two reviewers independently assessed the articles for quality and bias, and extracted the relevant information.

**Results:**

We identified 26 studies meeting the inclusion criteria. Pregnant women spent more than 50% of their time in sedentary behaviours. Increased time in sedentary behaviour was significantly associated with higher levels of C Reactive Protein and LDL Cholesterol, and a larger newborn abdominal circumference. Sedentary behaviours were significantly higher among women who delivered macrosomic infants. Discrepancies were found in associations of sedentary behaviour with gestational weight gain, hypertensive disorders, and birth weight. No consistent associations were found between sedentary behaviour and other variables such as gestational diabetes. There was considerable variability in study design and methods of assessing sedentary behaviour.

**Conclusions:**

Our review highlights the significant time spent in sedentary behaviour during pregnancy, and that sedentary behaviour may impact on pregnancy outcomes for both mother and child. The considerable heterogeneity in the literature suggests future studies should use robust methodology for quantifying sedentary behaviour.

**Electronic supplementary material:**

The online version of this article (doi:10.1186/s12966-017-0485-z) contains supplementary material, which is available to authorized users.

## Background

Sedentary behaviours are activities that expend very low energy, close to the basal metabolic rate, without significantly increasing energy expenditure. This equates to activities such as sitting or lying, that utilise less than 1.5 metabolic equivalent units, or times the basal metabolic rate [1, 2]. Sedentary behaviours are thus distinct from lack of physical activity, although the latter is sometimes mistakenly used as a marker of sedentary behaviour in the literature [3].

Epidemiological studies have shown that in the general adult population, around 55 to 60% of time awake is spent in sedentary behaviours [4, 5]. In the UK, children, young people, adults and older adults, spend on average at least half of their waking hours being sedentary [6, 7]. In pregnant women the situation appears to be similar or even worse [8–12], although the literature has not been systematically reviewed.

The quantity of time spent in sedentary behaviours is a key risk factor for diseases such as type 2 diabetes [13], cardiovascular disease [14], metabolic syndrome [15] and death from all causes [14, 16, 17]. New evidence also suggests that sedentary behaviour has an adverse effect on mental wellbeing, including depression [3]. Importantly some studies have exposed that even when individuals meet physical activity recommendations, they are still at a higher risk of premature death and adverse metabolic health if they sit for extended intervals [2, 18–20]. Sedentary behaviours, mostly television watching, are also linked to high risk of obesity and type 2 diabetes in the general population, independent of physical activity levels [1, 20], and in some studies the associations between sedentary behaviours with type 2 diabetes and with impaired glucose tolerance were stronger for women than for men [18, 21, 22].

If the negative health outcomes associated with sedentary behaviour in the general population, also occur in pregnancy, this could have implications for development of cardiometabolic complications such as gestational weight gain, gestational diabetes mellitus and hypertension, as well as mental wellbeing. It is not known whether sedentary behaviour in pregnancy influences outcomes for the baby such as birthweight or gestation at delivery.

We aimed to carry out a systematic review of the literature investigating sedentary behaviours during pregnancy to determine:

- the time spent in sedentary behaviours and the prevalence of sedentary behaviours among pregnant women, and
- whether sedentary behaviours are associated with pregnancy outcomes in mothers and offspring.

## Methods

### Data sources and searches

The Meta-analysis of Observational Studies in Epidemiology (MOOSE) guidelines were followed for the conduct [23], and the Preferred Reporting Items for Systematic Reviews and Meta-analyses (PRISMA) guidelines for the reporting of this systematic review [24]. The systematic review was registered in PROSPERO with the number CRD42015023611.

**Fig. 1:**
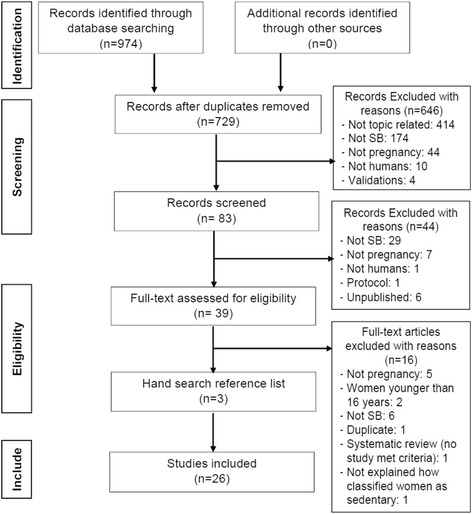
Search strategy flow diagram

Two researchers (CF, KL) independently performed the literature search using 5 different electronic bibliographic databases: MEDLINE, EMBASE, Web of Science, CINAHL and SPORTDiscus. The strategy (Fig. 1) was developed using Boolean. In MEDLINE medical subject headings (MeSH) used were: pregnant women (used also for pregnant woman), pregnancy (used also for pregnancies and gestation), prenatal care and sedentary lifestyle (used also for sedentary lifestyles). In EMBASE, main terms used were: pregnant woman (used also for pregnant women), pregnancy (used also for child bearing, childbearing, gestation, gravidity, intrauterine pregnancy, labour presentation, pregnancy maintenance and pregnancy trimesters), prenatal care (used also for ante natal care, antenatal care and antenatal control), prenatal period (used also for antenatal period) and sedentary lifestyle (used also for sedentary life style). The following keywords were also used for plain text searching in all databases: pregnan*, gestation*, gravid*, antenatal, prenatal, sedentar*, sitting, television, screen-based, TV, watching and viewing. Recursive searching of reference lists of retrieved articles was performed to identify any additional studies (Additional file 1).

Studies were included if the sample considered pregnant women over 16 years old, and if sedentary behaviours (specified as watching TV, sitting or lying, low energy expenditure activities, etc.) were assessed at any point during gestation. Only published studies were included. There were no exclusions related to study design, language, ethnicity, socioeconomic status, parity or physical condition.

Two reviewers (CF, KL) independently assessed articles for inclusion according to the inclusion/exclusion criteria. After screening the titles and abstracts, the reviewers selected potentially relevant studies. If it was not possible to determine relevance from titles and abstracts, full texts were retrieved. Any disagreements that could not be resolved by consensus were discussed with a third reviewer.

Two reviewers (CF, KL) independently extracted relevant information on study characteristics, methodology, and study results using a data extraction form in order to determine whether the study reported the time that pregnant women spent in sedentary behaviours, the prevalence of sedentarism among pregnant women, and whether the sedentary behaviours were linked to pregnancy outcomes.

For presentation in the tables reporting time and proportion of time in sedentary behaviours, we standardised the outcomes (converted to the same units) in order to make them comparable. Due to the heterogeneity of outcome data, a narrative synthesis was developed.

Quality and risks of bias were assessed using objective criteria relating to sample population and recruitment, reliability of instruments, use of validated outcome measures, follow-up, risk of bias and data analysis, using a quality assessment instrument that was modified from the Grading of Recommendations Assessment Development and Evaluation (GRADE) Guidelines used in assessment of clinical trials [25–28]. A paper could attain a maximum score of 8, a score of 1–3 indicating poor quality, 4–6 intermediate, and 7–8 good quality.

## Results

From 974 abstracts, 39 full text articles were assessed and 26 studies met the inclusion critera for the systematic review (Fig. 1).

### Characteristics of included studies

Characteristics of the 26 included studies are displayed in Table 1. Seventeen were cohort studies [8, 12, 29–43], 7 were cross-sectional studies [9–11, 44–47], and 2 were randomised controlled trials [48, 49].

**Table 1:** Description of included studies (arranged alphabetically)

Author	Country	Number of participants	Study design	Criteria for inclusion	Assessment method		Definition of sedentary	Quality
Both, et al. (2010) [31]	UK	11759	Cohort	Pregnant women due to deliver between April 1st 1991 and December 31st 1992.	Self-reported questionnaire.	Non-objective.	Who declared being mostly sitting.	Intermediate
Chasan-Taber, et al. (2014) [40]	USA	1276	Cohort	Women of Puerto Rican or Dominican Republic heritage.	Modified version of the Pregnancy Physical Activity Questionnaire (PPAQ).	Non-objective.	Activities expending <1.5 METs.	Intermediate
Chasan-Taber, et al. (2015) [34]	USA	1240	Cohort	Women of Puerto Rico or Dominican Republic heritage.	PPAQ.	Non-objective.	The sum of the MET-h/day spent watching TV/videos or sitting/standing at home, work, or during transportation.	Intermediate
Di Fabio, et al. (2015) [8]	USA	46	Cohort	Healthy pregnant women, including women between 18 and 45 years of age and singleton pregnancy.	- 7 day record diary.	- Non-objective.	Activities expending ≤1.5 METs (independent of nighttime sleep).	Intermediate
					- SenseWear® Mini armband accelerometer.	- Objective.		
					- ActivPAL™ Multi-sensor accelerometer.	- Non-objective.		
Evenson, et al. (2010) [37]	USA	1280	Cohort	Pregnant women ≥16 years of age.	The Behavioral Risk Factor Surveillance System (BRFSS).	Non-objective.	Two questions on TV watching and computer usage outside of work hours were used as SB indicators. Women were also asked if they were ‘mostly sitting’ during their usual daily activities.	Poor
Evenson, et al. (2011) [11]	USA	359	Cross-sectional	Pregnant women ≥16 years.	ActiGraph accelerometer.	Objective.	Activities expending <100 counts per minute.	Intermediate
Gollenberg, et al. (2010) [36]	USA	1006	Cohort	Latina ethnicity, age 16–40 years old, singleton pregnancy, and no prior participation in the study.	Modified version of the Kaiser Physical Activity Survey (KPAS).	Non-objective.	Hours spent TV watching per day and frequency of sitting at work.	Intermediate
Gradmark, et al. (2011) [47]	Sweden	101	Cross-sectional	Normal weight and overweight women without diabetes were studied.	Actiheart monitor.	Objective.	Epochs with valid heart rate data and zero accelerometry counts/min.	Intermediate
Hawkins, et al. (2014 Im.) [48]	USA	260	Randomized controlled trial	Women in their first trimester of pregnancy, between 16 and 40 years old, and at high risk for GDM.	Pregnancy Physical Activity Questionnaire (PPAQ).	Non-objective.	The amount of time spent watching TV or videos, or sitting or standing at home, work, or during transportation.	Good
Hawkins, et al. (2014 PA) [10]	USA	294	Cross-sectional	Women in the 2003–2006 NHANES study cycles who self-reported currently being pregnant, were 16 year or older, and who had available data on C reactive protein, physical activity, and SB.	ActiGraph accelerometer.	Objective.	Activities expending <100 counts per minute.	Intermediate
Hayes, et al. (2014) [49]	UK	183	Randomized controlled trial	All obese (BMI ≥ 30 K/m^2^) pregnant women.	- Acti-Graph accelerometer.	- Objective.	- Accelerometry: any minute with ≤100 counts/min.	Good
					- Recent Physical Activity Questionnaire (RPAQ).	- Non-objective.	- RPAQ, minutes spent on activities <1.5 MET.	
Hegaard, et al. (2010) [32]	Denmark	4558	Cohort	Danish-speaking pregnant women.	Self-reported questionnaires.	Non-objective.	Those who chose “mostly sitting” to describe most correctly her level of leisuretime activity.	Intermediate
Hegaard, et al. (2011) [35]	Denmark	4718	Cohort	Age ≥ 18 years, Danish speaking, singleton pregnancy, and intended spontaneous vaginal delivery.	Self-administered questionnaire.	Non-objective.	Those who answered: “Reading, watching television, or pursuing some other sedentary occupation”, as the most appropriate description of her activities.	Intermediate
Hjorth, et al. (2012) [9]	Ethiopia	304	Cross-sectional	All pregnant women who attended routine visits at the antenatal care clinic.	- Actiheart (heart rate and movement device).	- Objective.	Energy expenditure ≤1.5 METs.	Intermediate
					- 24 h physical activity recall.	- Non-objective.		
Jiang, et al. (2012) [30]	China	862	Cohort	Pregnant women over 20 years old in a singleton pregnancy, and had no disease including gestational diabetes (GD), hypertension, heart disease, chronic renal disease, and other diseases restricting physical activity.	Pedometer.	Objective.	Less than 5000 steps per day.	Intermediate
Kamareswaran, et al. (2013) [41]	UK	10	Cohort	Type 1 diabetes, current insulin pump therapy, and a viable singleton pregnancy.	Actiheart (heart rate and movement device).	Objective.	Activities expending ≤1 MET.	Intermediate
Li & Zhao (2007) [46]	China	405	Cross-sectional	Pregnant women working in a sewing factory.	Self-reported questionnaire.	Non-Objective.	According to the job, women were assigned to the study group (persistent sedentary) or control group.	Poor
Loprinzi, et al. (2013) [44]	USA	206	Cross-sectional	All women who answered the 2003–2006 National Health and Examination Survey.	ActiGraph accelerometer.	Objective.	Activity counts between 0 and 99 counts/min.	Intermediate
Lynch, et al. (2012) [43]	USA	1355	Cohort	Women from the ambulatory obstetric practices at ≤20 weeks of gestation.	PPAQ.	Non-objective.	The amount of time spent watching TV or videos, or sitting or standing at home, work, or during transportation.	Intermediate
Oken, et al. (2006) [29]	USA	1581	Cohort	Women attending initial prenatal visit, who delivered live infants.	Modified version of the leisure time activity section of the Physical Activity Scale for the Elderly (PASE).	Non-objective.	Hours per week spent watching TV or videos.	Intermediate
Padmapriya, et al. (2015) [42]	Singapore	1171	Cohort	Pregnant women aged 18 years and above attending first trimester antenatal dating ultrasound scan clinics.	Interview questionnaire.	Non-objective.	Hours spent on sitting plus hours spent on watching TV per day.	Intermediate
Reid, et al. (2014) [39]	Northern Ireland	100	Cohort	Healthy women, ≥16 years old, with singleton pregnancies, between 26 and 37 week gestation.	Body-media SenseWear Pro3 armband.	Objective.	Activities expending ≤1 MET.	Intermediate
Rhodes, et al. (2014) [33]	Canada	157	Cohort	The cohorts were couples without children, first-time parents during the first year of their parenthood experience, and second time parents during the first year of this parenting experience between the ages of 25 and 40 years of age.	GT1M Activity Monitor (accelerometer and stepcounter).	Objective.	Activities expending 0–100 average acceleration counts/min.	Intermediate
Ruifrok, et al. (2014) [12]	Netherlands	111	Cohort	Healthy pregnant women. Trial 1: nulliparous pregnant women without BMI restrictions, able to read, write and speak Dutch, and within their first 14 weeks of pregnancy; Trial 2: overweight and obese pregnant women at risk for gestational diabetes.	ActiTrainer accelerometer (Acti-Graph).	Objective.	Activities expending <100 counts/min.	Intermediate
Van Raaij, et al. (1990) [38]	Netherlands	18	Cohort	Healthy women judged by medical histories, blood pressure, hemoglobin concentration, and urine analysis.	- Open-circuit indirect calorimetry.	- Objective.	Lying, sitting quietly or very light sitting activity, or light-to-moderate sitting activity.	Intermediate
					- Physical activity diaries.	- Non-objective.		
Watts, et al. (2013) [45]	Australia	81	Cross-sectional	Pregnant women regardless of their pregnancy trimester.	The Australian Women’s Activity Survey (AWAS).	Non-objective.	Frequency and duration of sitting behavior.	Poor

Most studies were carried out in the USA (*n* = 11) and Europe (*n* = 9), and the remaining were in China (*n* = 2), Africa (*n* = 1), Canada (*n* = 1), Australia (*n* = 1) and Singapore (*n* = 1). One study included couples (for the purpose of this review we only considered data from the women, not the men) [33]; 2 other studies included both pregnant and non-pregnant women (non-pregnant women were considered in this review when comparisons between the two groups were made) [33, 47]. Three studies were conducted in Hispanic pregnant women [34, 40, 43], and 1 in Latina pregnant women [36]. One study was conducted in nulliparous pregnant women, 1 in obese pregnant women [49], 1 in pregnant women with type 1 diabetes mellitus [41], and 1 in pregnant women with sedentary lifestyles [38]. Thirteen studies utilised objective methods to assess sedentary behaviours (accelerometers, pedometers, combined heart rate and accelerometer device, and indirect calorimetry), and 13 studies employed non-objective measures including 4 administrating the Pregnancy Physical Activity Questionnaire (PPAQ), 9 using another kind of survey or questionnaire (The Australian Women’s Activity Survey, Modified version of the Kaiser Physical Activity Survey, Behavioral Risk Factor Surveillance System, modified version of the leisure time activity section of the Physical Activity Scale for the Elderly, and other type of non-objective appraisal methods) (Table 2). The PPAQ has been validated among pregnant women, similarly 2 of the administered surveys were also validated among pregnant women, meanwhile 3 studies used validated questionnaires, but not validated among pregnant women. Finally, 4 of the questionnaires were not validated.

**Table 2:** Characteristics of included studies

	Number of studies	Participants (N)
Assessment tool		
Accelerometer	7	1356
Accelerometer and HR sensor	3	415
Pedometer	1	862
Other objective	2	118
Pregnancy Physical Activity Questionnaire (PPAQ)	4	4131
Other self-reported	9	26559

### Amount and proportion of time spent in sedentary behaviours (Table 3)

**Table 3:** Time and proportion of time spent in sedentary behaviours

	Studies	N	Mean or median (SD or SE or IQR)
Time spent in SB (objective)			
Time spent in SB (h/day)	Ruifrok 2014 [12]	111	8.6 (SD 2.86)
	Hawkins 2014 [10]	294	9.2 (SE 16.2)^a^
	Loprinzi 2013 [44]	206	7.7 (SE 0.2)^a^
	Hjorth 2012 [9]	304	18.3^a^ (IQR16.65–19.6)
	Evenson 2011 [11]	359	7.07 (SE 0.165)^a^
	Di Fabio 2015 [8]	46	12.65 (SD 1.95)^a^
Sitting quietly or very light sitting activities (h/day)	Van Raaij 1990 [38]	18	6.7 (SD1.6)^a^
Light to moderate sitting activities (h/day)	Van Raaij 1990 [38]	18	1.6 (SD1.1)^a^
Sit/lie time (h/day)	Di Fabio 2015 [8]	46	18.2 (IQR17.1–19) w18; 18.3 (IQR17.6–19.4) w35
Time spent in SB (non-objective)			
Television time (h/day)	Padmapriya 2015 [42]	1171	2.4 (SD1.5)^a^
Total sitting time (h/day)	Padmapriya 2015 [42)	1171	8.6 (SD3.3)^a^
Proportion of time spent in SB (objective)			
% of day spent in SB	Hjorth 2012 [9]	304	76.4% (IQR 69.37–81.6^a^)
% of wear time spent in SB	Ruifrok 2014 [12]	111	65%
	Evenson 2011 [11]	359	57.1% (SE 0.77)
	Hawkins 2014 [10]	294	64.4% (SE 0.02)^a^
% of time awake in SB	Di Fabio 2015 [8]	46	76% (SD11) w18–78% (SD13) w35
% of day time in sit/lie	Di Fabio 2015 [8]	46	76% (IQR71–79) w18; 76% (IQR73–81) w35

^a^ Numbers were calculated as means and converted to the same units

The amount of time spent in sedentary behaviours was estimated in 8 studies using either objective [8–12, 30, 38, 44] or non-objective methods [42] (Table 3).

The time spent in sedentary behaviours during pregnancy assessed objectively, varied between 7.07 and 18.3 h per day. Of these studies 1 declared that sleeping was included [9], 2 stated that sleep time was not considered [8, 11], and the rest did not declare anything regarding sleep [10, 12, 44]. Meanwhile the study which assessed using a questionnaire found that women spent 2.4 h per day watching television and the mean of total sitting time was 8.6 h per day [42] (Table 3).

Among the 5 studies assessing the proportion of time spent in sedentary behaviours all used objective devices, finding that pregnant women spent more than 50% of their time (range 57.1 to 78%) in sedentary activities [8–12] (Table 3).

### Definitions of sedentary behaviours

The definition of time spent in sedentary behaviours differed according to method of assessment. Studies that used accelerometers defined activities with less than 100 counts per minute as sedentary behaviours, while activities expending 1.5 metabolic equivalents or less was used for combined heart-rate and activity monitors. Meanwhile, non-objective methods focused mostly on television viewing and sitting time.

### Prevalence of sedentarism among pregnant women (Table 4)

**Table 4:** Prevalence of sedentarism among pregnant women

Sedentary activity definition	Studies	Assessment method	N	Prevalence
Sedentary	Jiang 2012 [30]	Objective	862	18%
	Hegaard 2011 [35]	Non-objective	4718	29%
Watching TV or videos 5 or more (h/day)	Evenson 2011 [11]	Non-objective	359	15.3%
Watching TV 2 or more (h/day)	Oken 2006 [29]	Non-objective	1581	34%
Watching TV 3 or more (h/day)	Padmapriya 2015 [42]	Non-objective	1171	31.9%
Mostly sitting during day	Evenson 2011 [11]	Non-objective	359	24%

Five studies determined the prevalence of sedentarism among the pregnant population, all except 1 [30] used non-objective methods to assess activity behaviour, and all used their own cut-offs to classify women as sedentary. Two used the term “sedentary”, defining this as <5000 daily steps [30] or considering women as ‘sedentary’ if they declared “watching television, or pursuing some other sedentary occupation” as the most appropriate description of their activities [35], respectively. One study focused on the second trimester of pregnancy and found that prevalence of sedentarism was 18% [30], the other study assessed women on the third trimester of pregnancy finding that 29% were sedentary [35]. Three studies analysed the prevalence of sedentary women, however these 3 studies did not use the term ‘sedentary’, but used different activity categories defined variously by the authors as: “watching television (for a certain amount of time)”, or being “mostly sitting”. One study found that 15.3% of the studied women watched television or videos for 5 or more hours per day [37], other study found that 34% viewed television 2 h or more per day [29], and the last one found that 31.9% watched television more than 21 h per week, i.e. about 3 h per day [42]. Additionally 1 of the studies found that 24% of women were “mostly sitting” during usual daily activities [37] (Table 4). Comparison of data was difficult due to different cut-offs to define sedentary behaviour and categorisation of sedentarism.

### Change in sedentary behaviour during pregnancy

Among the included studies, 5 aimed to determine whether time spent in sedentary behaviours was stable or changed during gestation [8, 10–12, 37]. Four of these studies examined minutes per day or percentage of day spent in sedentary activities based on objective measures [8, 10–12]. Of these, only 1 found that the percentage of time awake spent in sedentary behaviours significantly increased between week 18 and 35 of gestation [8]. Another study found that women spent a mean of 40 min (standard deviation ±75) less in “very light sitting activities” (activities that spend around 1.3 times the basal metabolic rate) in later gestation than in earlier gestation [38]. The 3 studies which objectively assessed time or percentage of time of monitored time spent in sedentary behaviours, did not find significant differences in time spent in sedentary behaviours between trimesters of gestation [10–12]. When focused on the number of sedentary pregnant women across gestation, more women were sedentary during the third trimester than during the second trimester (18%, *n* = 155; 24.9%, *n* = 215, respectively) [30]. When the time spent between trimesters in TV watching and computer use was compared, no differences were found [37].

Five studies compared sedentary behaviours between pregnant and non-pregnant women [35, 38, 42, 43, 47]. Four compared from before pregnancy to during pregnancy, and 1 compared pregnant women versus one year postpartum women [38]. Three studies used non-objective methods [35, 42, 43], and 2 objective procedures [38, 47] to assess sedentary behaviours. All found that the time spent in sedentary activities is significantly greater among pregnant than non-pregnant women.

When the number of women that watched television for long periods was compared before and after pregnancy, 1 study observed that the number increased [42], and the other found no change [29].

### Additional factors affecting sedentary lifestyles

Some studies considered additional factors which could influence the development of sedentary lifestyles. These factors included: smoking, meeting physical activity recommendations, parity, maternal age, and education level. Time spent in sedentary behaviours was significantly less among women who smoked cigarettes in the past 5 days, compared to those who did not [11]. Time spent in sedentary behaviours at 35 weeks of gestation was significantly less among women meeting physical activity guidelines compared to women who did not [8]. During pregnancy women expecting their first child decreased their sedentary time significantly more than non-pregnant women without children, as well as first time pregnant women also decreased their sedentary time significantly more than those expecting their second baby as pregnancy advanced [33]. When the changes before and during gestation were compared, women aged 16–19 years, significantly decreased their sedentary activity compared to those aged 20–24 years. Women who had completed college, also significantly decreased their sedentary activity during pregnancy, compared with those with less than a high school education [43].

### Interruptions during sedentary time

One study focused on the transitions between sit to stand, using an objective device that evaluates postural allocation [8]. No differences were found in sit/lie and upright time between week 18 and 35 of gestation. However, the number of transitions between sedentary (sit/lie) to upright per day and the number of sit/lie bouts increased significantly from week 18 to week 35 of gestation, whilst the length of sit/lie bout in minutes per day significantly decreased across this gestation window.

### Associations between sedentary behaviours and maternal and infant outcomes

Birth and gestation outcomes associated with sedentary behaviours were studied in 14 of the included studies [10, 12, 30–32, 34, 36, 39, 40, 44–47, 49]. Of these, 7 were focused on pregnancy outcomes including gestational weight gain (GWG) and maternal depression [12, 30, 34, 40, 44–46], 5 on metabolic outcomes [10, 36, 44, 47, 49], and 5 on infant outcomes [12, 31, 32, 39, 49].

### Associations between sedentary behaviours and pregnancy outcomes (Table 5)

**Table 5:** Associations between sedentary behaviours and maternal health outcomes

	Author	Participants	Association (Yes/No)
Pregnancy Outcomes			
GWG	Ruifrok 2014 [12]	111	No
	Chasan-Taber 2014 [40]	1276	No
	Jiang 2012 [30]	862	Yes^a^ (*p* < 0.001)
Hypertensive disorders	Chasan-Taber 2015 [34]	1240	No
	Loprinzi 2013 [42]	206	No
	Li 2007 [46]	405	Yes^b^ (*p* < 0.05)
Depression	Watts 2013 [45]	81	No
Metabolic Outcomes			
Glucose levels	Loprinzi 2013 [44]	206	Trend (*p* = 0.06)
	Hayes 2014 [49]	183	No
Insulin sensitivity	Gradmark 2011 [47]	101	No
GDM	Hayes 2014 [49]	183	No
AGT	Gollenberg 2010 [36]	1006	No
CRP	Loprinzi 2013 [44]	206	Yes^c^ (*p* < 0.05)
	Hawkins 2014 [10]	294	Yes^c^ (*p* < 0.05)
Blood lipids levels (Total Cholesterol, HDL-cholesterol and triglycerides)	Loprinzi 2013 [44]	206	Yes^d^ (LDL *p* < 0.05)
Infant outcomes			
Birth Weight	Ruifrok 2014 [12]	111	No
	Hegaard 2010 [32]	4558	No
	Both 2010 [31]	11759	Yes^e^ (*p* < 0.05)
Macrosomia	Reid 2014 [39]	100	Yes^f^ (*p* < 0.05)
New-born abdominal circumference	Hayes 2014 [49]	183	Yes^g^ (*p* < 0.05)
Gestational length	Ruifrok 2014 [12]	111	No
	Both 2010 [31]	11759	No
Risk of preterm delivery	Both 2010 [31]	11759	No

^a^ GWG was higher in the sedentary group compared with the active group, ^b^ the sedentary group developed more hypertension, ^c^ Increased time in sedentary behaviours is associated with higher levels of CRP, ^d^ increased time in sedentary behaviour is associated with higher LDL cholesterol, ^e^ Increased time in sedentary behaviour is associated with lower birth weight, ^f^ women delivering macrosomic infants had higher levels of SB, ^g^ the association between SB and new-born abdominal circumference was inverse at baseline, and positive at 36 weeks

Three studies investigated whether there is an association between sedentary behaviours and gestational weight gain [12, 30, 40]. One study found no association between percentage of time spent in sedentary behaviours with gestational weight gain at 15 weeks of gestation, between 15 and 32–35 weeks of gestation, or with gestational weight gain per week [12]. Likewise, change in percentage of time in sedentary behaviours during 15 to 32–35 weeks of gestation was not associated with total gestational weight gain or with gestational weight gain per week. Another study also observed no significant associations between sedentary activity and inadequate or excessive gestational weight gain, at each stage of pregnancy [40]. However, in another study the ‘Active’ group (named according to author´s categorisation) gained significantly lower maternal weight during the second and third trimesters than the ‘sedentary’ group (named according to author´s categorisation) [30].

Three studies explored the association between pregnancy sedentary behaviours and hypertensive disorders during gestation. Two studies found no association [34, 44], but 1 study found that women who had persistent sedentary work (and were not authorised to move from their work place during working hours), such as sewing operators, developed significantly more gestational hypertension than women in the control group, whose work was also mostly sedentary, but whom were allowed to move during working time, such as secretaries [46].

No association was found between pregnancy sedentary behaviours and depression [45].

### Associations between sedentary behaviours and metabolic outcomes (Table 5)

The relationship between time spent in sedentary behaviours and fasting glucose levels was analysed in 1 study, finding a positive association [44]. On the other hand, sedentary behaviours were not associated with altered insulin sensitivity [47], gestational diabetes mellitus [49], or abnormal glucose tolerance [36]. Two studies found associations between sedentary behaviours and C-reactive protein (CRP) [10, 44]. In 1 study sedentary time and proportion of wear time spent sedentary were positively associated with CRP among women in the second trimester, but this finding was no longer statistically significant in analyses adjusting for confounders [10]. In the other study the positive association between sedentary behaviours and CRP levels remained after adjustment for confounders [44]. A significantly positive association between time spent in sedentary behaviours and higher LDL cholesterol was found in 1 study, but no association was found with any other blood lipid marker [44].

### Associations between sedentary behaviours and infant outcomes (Table 5)

Two studies found no association between birth weight and mother’s sedentary behaviours during pregnancy [12, 32]. One study found a significant association between lower birthweight with time spent in sedentary lifestyle in each trimester of gestation [31], whilst another found that women who delivered macrosomic infants (birthweight ≥4000 g) spent significantly more time sedentary than women delivering offspring weighing less than 4000 g [39]. The 1 study exploring the correlation between the new born abdominal circumference (as an indicator for abdominal adiposity) with mothers’ time spent sedentary found differing results according to gestation. At 16–18 weeks of gestation a significantly inverse association was found between infant abdominal circumference and time spent sedentary, however at 36 weeks of gestation, the relationship became significantly positive [49]. No associations were found between sedentary behaviours and gestational length [12, 31], or risk of preterm delivery [31].

### Quality assessment results

Both reviewers agreed that 2 (7.7%) of the studies were of good quality [48, 49], 3 (11.5%) were classified as of poor quality [37, 45, 46], and the rest 21 as intermediate (80.8%).

The 2 studies that were classified as good quality were randomised controlled trials.

Of those classified as poor quality the main reasons were small sample size [45, 46], use of a non-objective appraisal tool to classify women as sedentary [37, 45, 46] and lack of detail about the outcome measures [37, 46].

## Discussion

### Main findings

There is increasing interest in research in the general population about whether reducing time spent in sedentary behaviours has a beneficial effect on health [50, 51]. Here we systematically reviewed the literature in this field among pregnant women. Our key findings were that pregnant women spend at least half of their time in sedentary activities, which is similar to time reported in children, young people, adults and older adults in the UK [6]. Whether sedentary behaviours impact on pregnancy outcomes was less clear-cut with inconsistencies in the literature.

Our review highlights the considerable heterogeneity in the definitions of sedentary behaviours and the methods used to assess this. Differences in the reported prevalence of sedentary behaviours between studies could be due to the unclear definition of sedentary behaviours, or classification of sedentary. For example, 1 study used a pedometer, an objective method, to classify women as sedentary, considering less than 5000 steps per day as a sedentary lifestyle [30], meanwhile in another study women were considered sedentary if they answered “Reading, watching television, or pursuing some other sedentary occupation”, as the most appropriate description of their activities during pregnancy [35]. Many of included studies defined sedentary behaviours as activities expending the same or less than one metabolic equivalent [39, 41], however there is no consensus in how many hours per day spent in sedentary behaviours are sufficient to be categorised as sedentary, making it difficult to determine the prevalence of sedentarism. In addition sedentary behaviours were often assessed retrospectively [32, 35], potentially introducing recall bias.

Studies also differed in the assessment measures to calculate sedentary behaviours making comparisons difficult. This corresponds with what has been exposed regarding sedentary behaviours assessment in other populations [6].

Half of the identified studies considered whether sedentary behaviour in pregnancy impacted on maternal or offspring outcomes. This is an important consideration as interventions based on increasing physical activity among obese pregnant women have had limited impact on pregnancy outcomes [49, 52–55]. One study found that reducing time spent in sedentary activity was associated with decreased gestational weight gain [30]. Two other studies, including a large study of >1000 women found no associations with gestational weight gain [12, 40]. Likewise there were discrepancies in studies examining associations of sedentary behaviours with hypertensive disorders [34, 44, 46]. Notably the 1 study which found a significant association was classified as poor quality, which decreases the reliability of the result [46]. Differences in ethnicity between the study populations may partly explain the discrepant findings with gestational weight gain (1 study developed in Denmark, other included only Latin-American pregnant women, and 1 was developed in China) and hypertensive disorders (1 included only Latin-American women, 1 was developed in the USA and 1 in China). No association was found between depression and sedentary behaviours, however the 1 study focusing on that was classified as poor quality [45]. None of the studies reported associations between sedentary behaviour and glucose metabolism, as assessed by fasting glucose levels [44, 49], insulin sensitivity (measured using an oral glucose tolerance test) [47], gestational diabetes mellitus (GDM) [49] and in a large study of >1000 women glucose tolerance measured during a glucose tolerance test [36]. In contrast, 2 studies found associations between higher CRP levels and increased sedentary behaviour [10, 44], and 1 found an association with blood lipids [44] suggesting there may be subtle beneficial effects on maternal metabolism if time spent sedentary is reduced. Overall, there was some suggestion that sedentary behaviours may impact on size at birth [31, 39, 49], but not timing of delivery [12, 31]. However, the largest study including over 11,000 pregnant women and which reported associations of sedentary behaviour with birthweight but not gestational length or risk of preterm birth, assessed sedentary behaviours during pregnancy using a postal questionnaire using the question “Are/were you mostly sitting?” [31].

### Strengths and limitations

The strengths of this review include the systematic and comprehensive review process which was followed in line with PRISMA guidelines. Two researchers independently assessed eligibility of the titles, abstracts and full-text studies, extracted the data and assessed the articles for bias.

A further strength of the review is that many of the studies were of considerable sample size. Eleven studies included samples of over 1000 women [29, 34, 36, 37, 40, 42, 43], including 2 assessing more than 4000 women using validated questionnaires [32, 35]. Nevertheless, larger studies using objective assessments of sedentary behaviour in pregnancy would considerably add to the literature in this field.

There are also some potential limitations. Though we used a robust search strategy developed from other systematic reviews of sedentary behaviour in the general population [2, 56, 57], it is possible that some potentially eligible studies may not have been identified. For example, some studies appraise sedentary behaviours when assessing physical activity, but the titles do not mention the key words we chose to identify sedentary behaviours. We included a search of reference lists of all papers that the full text was read, to identify any further additional papers.

A limitation of the data is that only 2 of the identified studies were trials, all the rest were observational. Of the trials, just 1 used an objective method to assess sedentary behaviours, the other employed a questionnaire. Of the 24 observational studies, only 12 used objective instruments, the other 12 utilised self-reported methods to assess sedentary behaviours. Most of these studies were considered of intermediate quality due to the small sample size, or lack of use of a validated questionnaire or objective measurement. Therefore, the use of objective methods, such as accelerometers, or the combination of movement and physiological (e.g. heart rate) devices should be encouraged if we wish to provide a more clear, realistic, and objective estimate of time spent in sedentary behaviours. Also, the cut-offs used for defining sedentary behaviours as to categorise people as sedentary are not clear and differ between studies, and should be standardised.

Although 3 studies (11.5%) were classified as poor quality one of these [37] did not report any maternal or infant outcomes and so will not have influenced our interpretation of the literature. As noted the findings of the other 2 poor quality rated studies [45, 46] should be interpreted with caution. The rest of the studies were classified at least as intermediate quality, mostly because the designs were less reliable (not randomised controlled trials), most of the sample size were small, some utilised non-objective assessment methods, and/or were not validated, but we are confident that they are representative of the available literature.

## Conclusions

The observation that pregnant women spend much of their time in sedentary activities opens new approaches aiming to improve pregnant women’s health. However our review has identified important gaps in our understanding in this field. For example only 2 studies considered sleeping time during pregnancy [8, 38] which may be an important consideration when assessing sedentary behaviour due to changing sleep patterns in pregnancy. Further, only 1 study assessed the transitions from sit/lay to stand, or breaks during sedentary time [8], which may be an important area to target in future interventions studies.

Our review highlights a high prevalence of sedentarism and significant time spent in sedentary behaviours, also that changes in sedentary behaviour may impact on pregnancy outcomes for both mother and child, emphasising this as an area for future mechanistic and intervention studies. However, the heterogeneity in the literature suggests future studies should use robust methodology, preferably with objective measures for quantifying sedentary behaviour.

## Additional file

Additional file 1: Database search strategy. (PDF 8 kb)

## Abbreviations

CF: Caterina Fazzi
CRP: C-reactive protein
GRADE: Grading of Recommendations Assessment Development and Evaluation
KL: Kathryn Linton
MeSH: Medical subject headings
MOOSE: Meta-analysis of Observational Studies in Epidemiology
PRISMA: Preferred Reporting Items for Systematic Reviews and Meta-analyses
