# Supplementary material for: Sedentary behaviours during pregnancy: a systematic review
Source: Int J Behav Nutr Phys Act. 2017 Mar 16;14:32. doi: 10.1186/s12966-017-0485-z (PMC5353895; doi:10.1186/s12966-017-0485-z)
Supplement: Additional file 1: — Database search strategy. (PDF 8 kb) [file 12966_2017_485_MOESM1_ESM.pdf]

Ovid MEDLINE (R) 1946 to October Week 3 2015			
#	Searches	Results	Search Type
1	Pregnant women (MeSH)	5528	Advanced
2	Pregnancy (MeSH)	733929	Advanced
3	Prenatal care (MeSH)	21779	Advanced
4	Sedentary lifestyle (MeSH)	4043	Advanced
5	pregnan* OR gestation* OR gravid* OR antenatal OR prenatal (keywords)	235431	Advanced
6	sedentar* OR sitting OR television OR screen-based OR TV OR watching OR viewing (keywords)	12222	Advanced
7	1 OR 2 OR 3 OR 5	759044	Advanced
8	4 OR 6	14885	Advanced
9	7 AND 8	**235**	Advanced

EMBASE 1980 to 2015 Week 42			
#	Searches	Results	Search Type
1	Pregnant woman (MeSH)	41562	Advanced
2	Pregnancy (MeSH)	549150	Advanced
3	Prenatal care (MeSH)	29123	Advanced
4	Prenatal period (MeSH)	7605	Advanced
5	Sedentary lifestyle MeSH)	6776	Advanced
6	pregnan* OR gestation* OR gravid* OR antenatal OR prenatal (keywords)	282991	Advanced
7	sedentar* OR sitting OR television OR screen-based OR TV OR watching OR viewing (keywords)	14853	Advanced
8	1 OR 2 OR 3 OR 4 OR 6	667010	Advanced
9	5 OR 7	20121	Advanced
10	8 AND 9	**285**	Advanced

Web of Science 30-10-2015		
#	Search	Results
1	TI= (pregnan* OR gestation* OR gravid* OR antenatal OR prenatal)	
2	TI= (sedentar* OR sitting OR television OR screen-based OR TV OR watching OR viewing)	
3	2 AND 1	**406**

CINAHL 30-10-2015		
#	Search	Results
1	TI (pregnan* OR gestation* OR gravid* OR antenatal OR prenatal) AND TI (sedentar* OR sitting OR television OR screen-based OR TV OR watching OR viewing)	**39**

SPORTDiscus 22-10-2015		
#	Search	Results
1	TI (pregnan* OR gestation* OR gravid* OR antenatal OR prenatal) AND TI (sedentar* OR sitting OR television OR screen-based OR TV OR watching OR viewing)	**9**
